# Deficiency of Formyl Peptide Receptor 1 and 2 Is Associated with Increased Inflammation and Enhanced Liver Injury after LPS-Stimulation

**DOI:** 10.1371/journal.pone.0100522

**Published:** 2014-06-23

**Authors:** Arne Giebeler, Konrad L. Streetz, Oliver Soehnlein, Ulf Neumann, Ji Ming Wang, Lars-Ove Brandenburg

**Affiliations:** 1 Department of Surgery, University Hospital RWTH Aachen University, Aachen, Germany; 2 Department of Anatomy and Cell Biology, RWTH Aachen University, Aachen, Germany; 3 Department Medicine III, University Hospital RWTH Aachen University, Aachen, Germany; 4 Institute for Cardiovascular Prevention (IPEK), LMU Munich, Munich, Germany; 5 Laboratory of Molecular Immunoregulation, Cancer and Inflammation Program, Center for Cancer Research, National Cancer Institute, Frederick, Maryland, United States of America; National Institutes of Health, United States of America

## Abstract

**Introduction:**

Formyl peptide-receptor 1 and 2 (FPR1 and FPR2) in mice were identified as receptors with contrary affinity for the PAMP fMLF. Formyl-methionyl-leucyl-phenylalanine is either part of the bacterial membrane and is secreted by the mitochondria of eukaryotic ceslls during apoptosis. Furthermore FPR1 and 2 are described as highly relevant factors for the chemotaxis of immune cells. Their role during the acute liver injury has not been investigated yet.

**Materials and Methods:**

Constitutive knockout mice for FPR1 (mFPR1^-/-^), FPR2 (mFPR2^-/-^) and wild type (WT) mice were challenged with LPS i.p. for 3 h and 6 h. Liver and serum were sampled for further analysis.

**Results:**

Liver transaminases were elevated in all mice 3 h and 6 h post LPS stimulation. Gene expression analysis displayed a reduced expression of the pro-inflammatory cytokines IL-6 and CXCL1 after 3 h in the mFPR1^-/-^ compared to wild type and mFPR2^-/-^ mice. After 6 h, IL-6, TNF-α and CXCL1 were significantly higher in mice lacking mFPR1 or 2. Consistent to these findings the numbers of CD11b^+^ and Ly6G^+^ immune cells were altered in the livers. The analysis of TLR2 and TLR4 revealed time and genotype specific changes in theirs gene expression. Additionally, the liver in mFPR1- and mFPR2-deficient mice seem to be more susceptible to apoptosis by showing a significant higher number of TUNEL^+^-cells in the liver than WT-mice and displayed less Ki67-positive nuclei in the liver.

**Conclusion:**

The results suggest a prominent role of FPRs in the regulation of the hepatic inflammatory response after LPS induced liver injury. Deletion of mFPR1 or mFPR2 leads to deregulation of the inflammatory response compared to WT mice, associated with more severe liver injury represented by higher levels of transaminases, apoptotic cells and a reduced regenerative capacity.

## Introduction

The inflammatory response after a liver injury is important for the induction of liver regeneration [1], [2]. Perturbation in mediating the inflammatory response leads to deregulation of the liver regeneration and finally to a higher degree of liver injury [1]–[3]. Failure in resolution of the injury stimulus leads to a chronic liver injury resulting in chronic liver diseases, e.g. liver fibrosis [3]. During the process of liver injury, parenchymal liver cells undergo apoptosis [4]. Among the process of apoptosis, small molecules mediating the cellular damage (Damage associated molecular patterns/DAMPs) are secreted to the physiological environment [5]. Among these DAMPs is a small group of molecules which are evolutionary prokaryotic origin. Those molecules are classified as the DAMP-subgroup Pathogen-associated-molecular patterns (PAMPs) [6]. In general PAMPs are functioning as an important molecule for recognition of pathogens such as bacteria by the innate immunity [6]. One of these secreted PAMPs is N-formyl-methionyl-leucyl-phenylalanine peptide (fMLF/fMLP) [7]. The molecule fMLF is known as an inducer of chemotaxis for neutrophil granulocytes and monocytes after cellular damage [5], [8]. So far, two sources for fMLF are known. First, the bacterial cell wall could be identified as a source [9]. Later the mitochondria were described as a second source for the secretion of fMLF [8]. The release of fMLF is directly related to cellular apoptosis [8]. Known receptors for the fMLF-peptide are the formyl peptide receptors (FPR). The FPRs belong to the family of G-protein coupled receptors. Up to now 3 members of the formyl-peptide receptor family are known. This family is an example for non-homology among receptor families. Sequence analysis of FPR1, FPR2 (FPRL1 in human) and FPR3 (FPRL2 in human) do show a similarity by 69% (FPR1 to FPRL1) and 56% (FPR1 to FPRL2) [10]. Furthermore FPR1 shows high affinity towards fMLF, whereas FPR2 is a low-affinity receptor for fMLF and only high concentrations of fMLF are able to activate its signalling pathways. The third receptor FPR3 (FPRL2) shows no affinity for fMLF at all [11], [12]. Also the distribution and the role of these receptors among tissues and cells are various. FPR1 is a relevant receptor for the chemotatic movement of neutrophils and monocytes [13]. Neutrophils with a deficiency for FPR1 displayed an unorientated movement towards a side of injury and failed to reach this area [5], [14]. Besides its presence on the surface of hematopoietic cells FPR1 and FPR2, as well as theirs murine analogs, is also present on the surface of various organs (Brain, Liver, Kidney, and Intestine) [15]–[17]. The second member of the FPR-Family, FPR2, also known as FPRL1/LipoxinA4-receptor is poorly chemotatic and only high concentrations of fMLF induce its signalling regarding to this PAMP [18]. Furthermore the signalling of both receptors is highly various and depends on the receptor-ligand interaction [19]. The role of bacterial translocation in liver diseases has changed in the last years. Being suggested as a late stage event [20], it was shown that early bacterial translocation is a main reason for the establishment of liver fibrosis [21] and the progress of liver injury and survival of the bacterial infection was furthermore linked to the bacterial burden [22].

These prior findings suggest a differential role of FPR in the recruitment of the different leucocyte subtypes and who might have different functions divided in between tissue resident and towards injury site recruited cells [23]. Despite the fact of their well understood role in the chemotatic movement of hematopoietic cells [5], [8], their role in parenchymal cells such as hepatocytes are poorly understood. Despite the knowledge that these receptors are both present in murine liver [17], little is known about their role during the acute bacterial induced hepatitis as well as their impact in acute liver diseases is not present in the literature yet. We performed a study using the LPS-model to induce an acute liver injury in wild type (WT) and constitutive mFPR1- and mFPR2-knockout mice. Afterwards we performed histological, clinical and biochemical analysis of the observed effects in the liver of those animals.

## Materials and Methods

### Animal experiments

Five 8-10 weeks old wild type, mFPR1^-/-^ [24], mFPR2^-/-^ [25] C57/Bl6 mice were stimulated with 4mg/Kg bodyweight *E. coli* LPS (Sigma-Aldrich, Steinheim, Germany) and kept for 3 h and 6 h. The mFPR1 Mice were a kind gift from Dr. Philip Murphy of the National Institute for Allergy and Infectious Diseases, NIH [24]. The mFPR2 knockout mice were generated as described previously [25]. Littermates were used as controls.

Mice were sacrificed 3 h and 6 h post LPS-stimulation, blood and liver were removed and preserved for biochemical and immunohistological assays. After stimulation the mice were kept in SII-long-cages with access to food and water ad libitum. Blood was taken retroorbital before sacrifice of the mice. The serum was separated by centrifugation and stored at −20°C until measurement.

All experiments were performed in accordance to the German protection of animals act and with permission of the authority of the federal state North Rhine Westphalia. The study protocol was approved by the institutional animal care and use committee (Landesamt für Natur-, Umwelt und Verbraucherschutz (LANUV), Duesseldorf, Reference number: 84-02.04.2013.A246).

### RNA-Isolation

Cryopreserved liver tissue was homogenized and RNA was extracted by using the Nucleospin RNA-II Kit (Macherey-Nagel, Dueren,Germany). Afterwards 400 ng of total RNA was converted into cDNA by using the Omniscript reverse transcriptase kit (Qiagen, Hilden, Germany). All proceedings were performed according to manufacturers' guidelines.

### Quantitative PCR assay

The gene expression analysis was performed using an ABI 7500 Real-Time PCR (Life Technologies, Darmstadt, Germany). Genexpression analysis for the murine (ms) genes IL-6, TNF-α, CXCL1, TLR2, TLR4, mFPR1, mFPR2 and mFPR3 were performed. The Primers for mFPR1 and mFPR2 were published previously [26]. Murine qPCR Primers were designed using the Primer Express 3.0 Software provided by Life Technologies (Darmstadt, Germany). Specific PCR products were detected by Sybr-Green and changes in gene expression were analysed by the ΔΔCT-calculation. GAPDH was used as a housekeeping gene (Table 1).

**Table 1:** qPCR Primer.

Gene	Sequence	Annealing temp-.
**IL-6 ms fw**	AGAAGGAGTGGCTAAGGACCAA	58°C
**IL-6 ms rv**	ACGCACTAGGTTTGCCGAGTA	
**CXCL1 ms fw**	CTAGTAGAAGGGTGTTGTGCGAAA	59°C
**CXCL1 ms rv**	AAACACAGCCTCCCACACATG	
**GAPDH ms fw**	TGTTGAAGTCACAGGAGACAACCT	58–60°C
**GAPDH ms rv**	AACCTGCCAAGTATGATGACATCA	
**TNF-α ms fw**	AGGACCCAGTGTGGGAAGCT	59°C
**TNF-α ms rv**	AAAGAGGAGGCAACAAGGTAGAGA	
**TLR2 ms fw**	CCCTTCTCCTGTTGATCTTGCT	58°C
**TLR2 ms rv**	CGCCCACATCATTCTCAGGTA	
**TLR4 ms fw**	GCAGAAAATGCCAGGATGATG	59°C
**TLR4 ms rv**	TCTGATCCATGCATTGGTAGGT	
**mFPR3 fw**	CCTTTGTTAATTCCAGCCGTCC	60°C
**mFPR3 rv**	TCTCTTTGAGCCAGACTGTGCC	

### Immunohistochemistry for immune cell marker

Formalin fixated paraffin embedded (FFPE) liver samples were cut into 5 µm strong sections and stained for CD11b (Abcam, Cambridge, UK, rabbit-anti-mouse) and Ly6G (Affimetrix-eBioscience, Frankfurt/Main, Germany, rat-anti-mouse). The primary antibody was used in a dilution of 1∶100 and a species specific secondary antibody with a HRP-conjugate was diluted 1∶500 to detect the primary antibody. Visualization was performed using 3,3′-diaminobenzidine tetrahydrochloride (DAB) (Sigma-Aldrich, Steinheim, Germany). Antigen retrieval was executed according to manufacturer's instruction. Nuclei were counterstained with Haematoxylin (Sigma-Aldrich, Steinheim, Germany). For each individual animal/genotype 7 pictures were taken in 100 and 200 fold magnification using an Olympus BX51. The 100 fold magnification was used for overview whereas the 200 fold magnification pictures were used for the detailed analysis and counting of the total CD11b^+^- or Ly6G^+^-cells per view field.

For the immunofluorescent staining of CD11b^+^-cells in the BDL-model, cryosections of liver tissue were made and stained with a CD11b-antibody (rat-anti-mouse, eBiosciences, Frankfurt/Main, Germany) used in a 1∶200 dilution. Visualization was performed using an anti-rat ALEXAFLUOR546 (Life Technologies, Darmstadt, Germany). Fluorescent microscopy pictures were taken by an AxioImager Z1 (Carl-Zeiss, Jena).

### Tunel staining

FFPE-liver tissue was cut as described above in 5 µm strong sections. The TUNEL-staining was made according to the manufacturers' instruction (Merck-Millipore, Darmstadt, Germany). Visualization was performed using DAB and Nuclei counterstaining was made with Haematoxylin (Sigma-Aldrich, Steinheim, Germany).

### Ki67-staining

The analysis of the ubiquitous cell cycle marker Ki67 was performed using the rat-anti mouse Ki67 (Tec3-clone, DAKO, Hamburg, Germany) in a 1∶50 dilution. The detection was conducted with a secondary, HRP-conjugated anti-rat antibody in a 1∶300 dilution. The visualization was performed with DAB (Sigma-Aldrich., Steinheim, Germany). Nuclei counterstaining was performed using Haematoxylin (Sigma-Aldrich Steinheim, Germany).

### Statistics

The datasets were analyzed using the Studentś T-test and p-values ≤0.05 were regarded as significant and indicated in the respective graph.

## Results

### FPR-deficiency leads to increment of clinical parameter of liver injury

**Figure 1:**
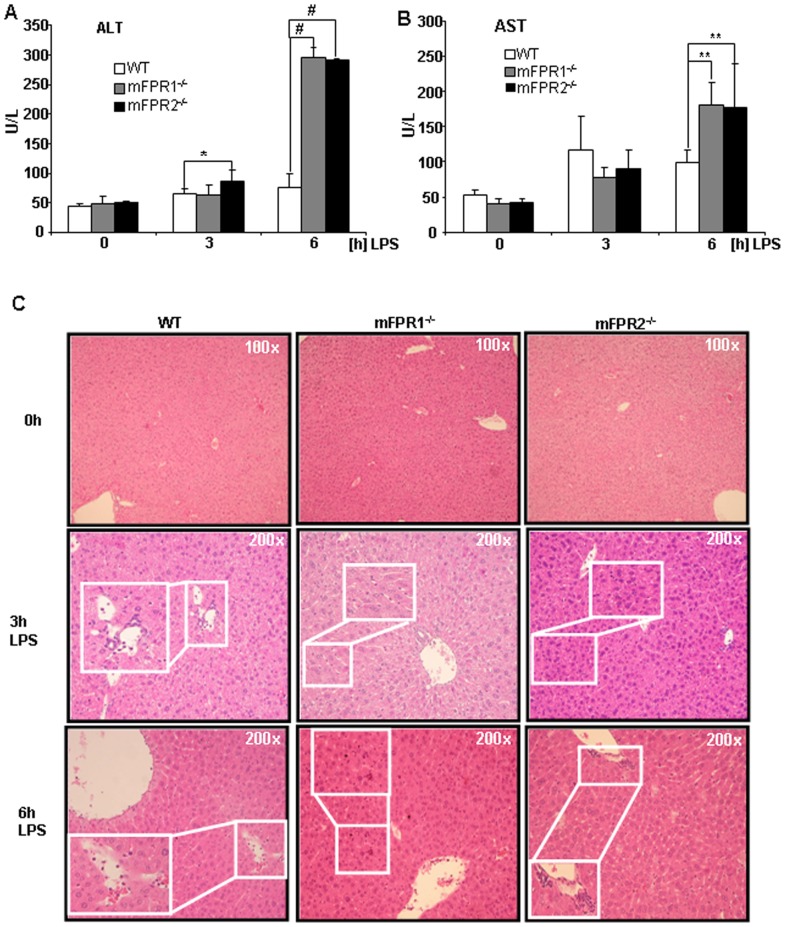
The clinical and histological analysis after LPS-stimulation includes measuring of ALT (A) and AST (B). The histological analysis was performed using H&E staining (C). Overview pictures at 0 h were taken in 100-fold magnification, detail microphotographs at 3 h and 6 h post LPS such as infiltrating immune cells are magnified 200-fold (* = p<0.05; *** = p<0.005).

Analyses of clinical parameters such as transaminases provide a quick overview about physiological condition in regard of liver injury. The alanine-aminotransferase (ALT) and aspartate-aminotransferase (AST) of WT, mFPR1^-/-^, mFPR2^-/-^ mice displayed no initial differences among the different genotypes. After LPS stimulation for 3 h the transaminases were significantly increased in mFPR2^-/-^ mice (86 U/L) in comparison to wild type mice (66 U/L), whereas the mFPR1^-/-^ mice displayed elevation after 3 h LPS administration, but did not reach a level of significant difference (63 U/L). The analysis of the 6 h time point displayed that transaminases were significantly upregulated in the serum derived from mFPR1^-/-^(296 U/L) or mFPR2^-/-^(291 U/L) mice compared to control (76 U/L) mice after LPS-treatment (Fig. 1A and 1B). The histological analysis of the livers by H&E staining after LPS-treatment showed an increased inflammatory response in the livers of all mice in concordance with the elevated levels of transaminases (Fig. 1C).

### Higher presence of inflammatory cells after LPS treatment

**Figure 2:**
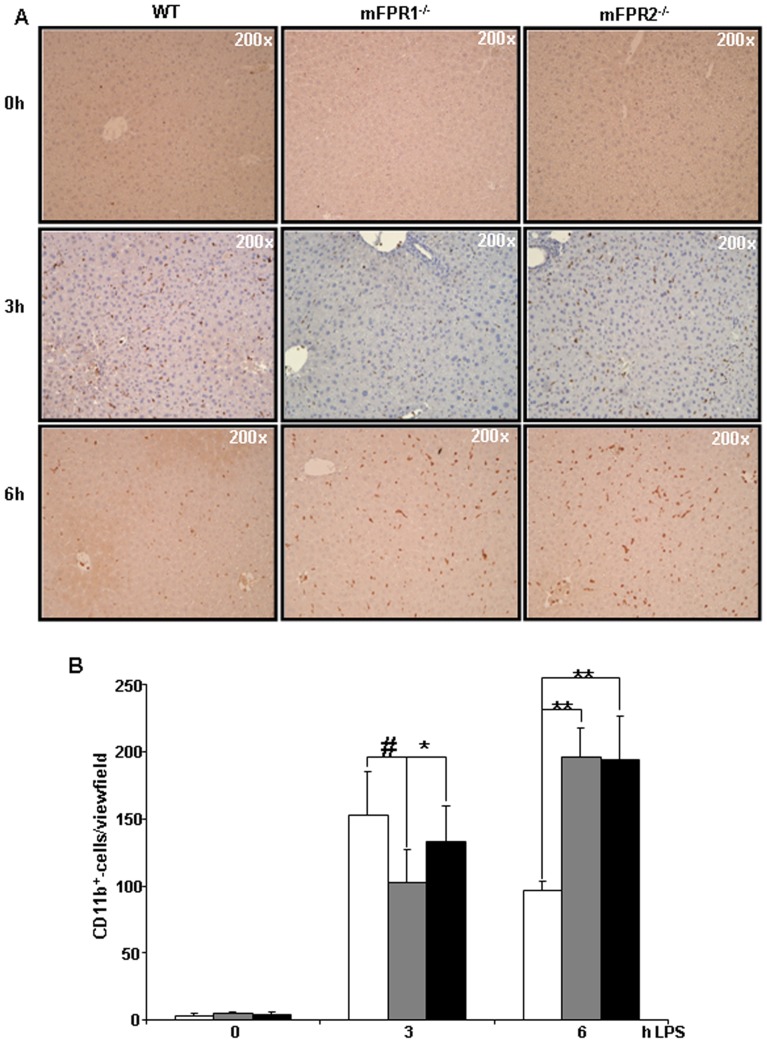
To identify the infiltrating immune cells after LPS stimulation according to their surface markers, CD11b was used to detect infiltrating monocytes and macrophages in the liver (A). Pictures were taken in 200-fold magnification and the CD11b^+^-cells were counted. The results were displayed as a graph (B) indicating differences among WT, mFPR1^-/-^ and mFPR2^-/-^ (* = p<0.05; ** = p<0.01; # = p<0.0001).

**Figure 3:**
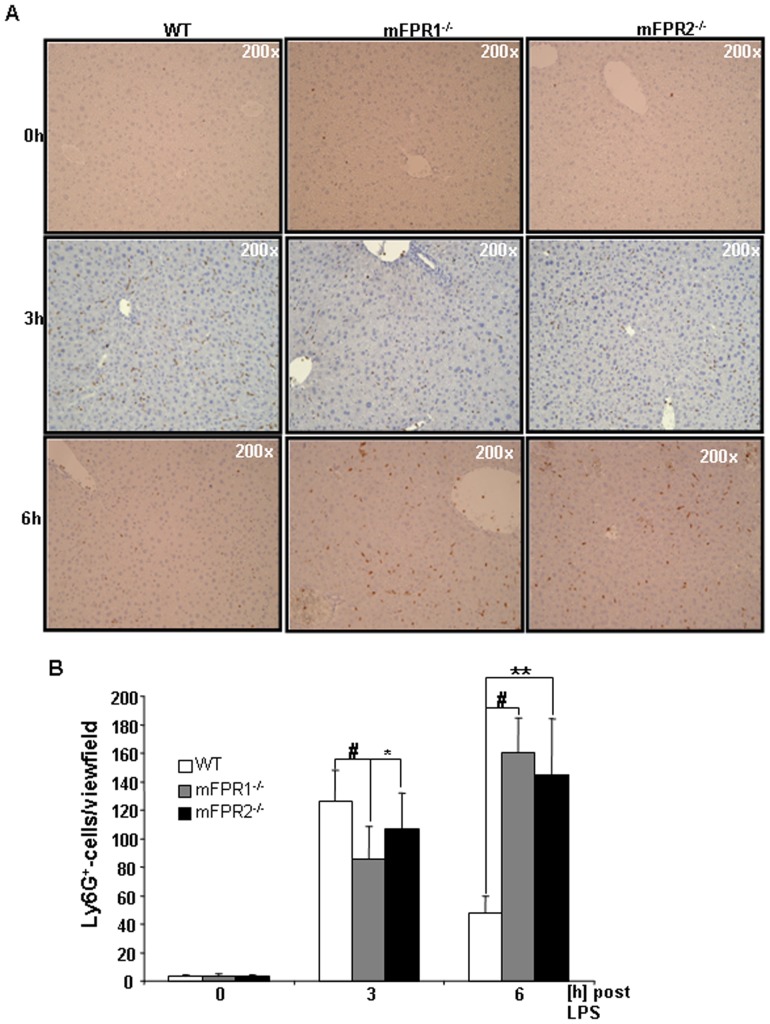
The second subset of immune cells were analysed by Ly6G-staining. Mainly neutrophil granulocytes were identified as Ly6G^+^ (A). Pictures were taken in 200-fold magnification and the Ly6G^+^-cells were counted. The results were displayed as a graph (B) indicating differences among WT, mFPR1^-/-^ and mFPR2^-/-^ (* = p<0.05; ** = p<0.01; # = p<0.0001).

In order to get a better overview of the different immune cell subtypes infiltrating into the liver, we performed immunohistological straining for the surface markers CD11b and Ly6G. These markers identify two relevant cells types involved in promotion of liver injury. CD11b is known as a marker for activated monocytes whereas Ly6G is known to detect neutrophil granulocytes specifically. The immunohistological analysis of CD11b and Ly6G displays a strong presence of both cell types at 3 h as well as 6 h post LPS-treatment. At the time point 3 h after LPS the mFPR1^-/-^ and the mFPR2^-/-^ mice displayed lower number of CD11b^+^(Fig. 2A and B) and Ly6G^+^-cells (Fig. 3A and B) in the liver compared to wild type. The difference between mFPR1^-/-^ mice and WT-mice was highly significant either among the CD11b^+^- (153±32 vs 102±24 (p<0,001)) and the Ly6G^+^-cells (WT126±21 vs mFPR1^-/^85±22 (p<0,001)). Also significantly lower were the CD11b^+^-cells (153±32 vs 133±26 (p<0,05)) and the number of Ly6g^+^-cells (126±21 vs 106±25 (p<0,05)) in the mFPR2^-/-^ mice in comparison to WT mice. At the later time point, 6 h after intraperitoneal application of LPS (Fig. 2A and B) the mFPR1- (196±21) and mFPR2-KO (194±34) mice displayed the higher number of CD11b^+^-cell infiltrates per view field in comparison to wild type mice (96±6). Likewise to the CD11b^+^-cells a higher presence of Ly6G^+^-cells was found in the livers of mFPR1^-/—^ (160±24) and mFPR2^-/—^mice (144±39; Fig. 3A and B). The numbers of the Ly6G^+^-cells in the wild type mice (47±11) were significantly lower (WT vs. mFPR1-KO p<0.001; WT vs. mFPR2-KO p<0.01). Taken together different degrees of inflammation occurred in regard to the deficiency of either mFPR1 or mFPR2 as wells as in regard to the temporal progression of inflammation.

### Increased Expression of pro-inflammatory genes

To analyse the inflammatory response further we performed qPCR analysis from whole liver extracts in order to investigate the expression of pro-inflammatory genes.

**Figure 4:**
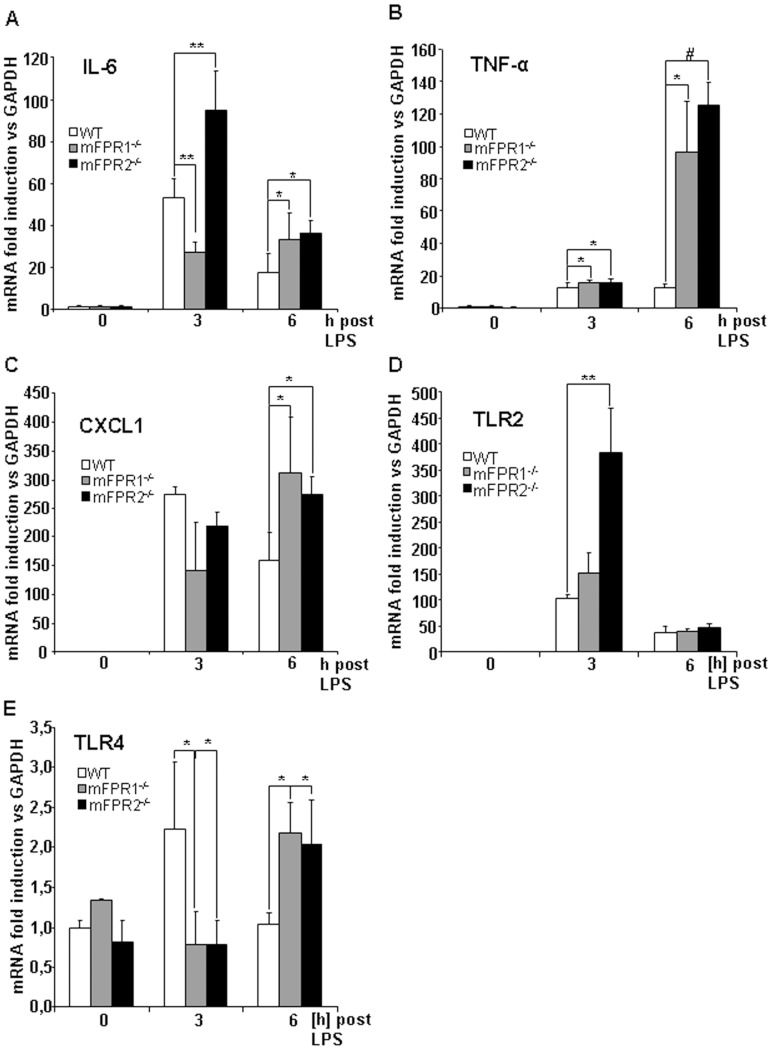
To better understand mechanism underlying the infiltration of immune cells into the liver of mFPR-deficient mice the gene expression of the pro-inflammatory cytokines IL-6 (A), TNF-α (B), CXCL1 (C), TLR2 (D) and TLR4 (E) were analyzed by qPCR. Changes in gene expression were related to GAPDH as a housekeeping gene. (* = p<0.05; ** = p<0.01; # = p<0.0001.)

The analysis of cytokine gene expression was contradictory between the two analysed time points. At 3 h post LPS injection an increased expression for the IL-6 gene could be detected in the WT mice compared to the mFPR1^-/-^ mice, who showed a significantly lower expression of IL-6 mRNA (p<0.01) whereas the mFPR2^-/-^ mice had a significantly higher expression compared to WT-mice (p<0.01). At 6 h post LPS injection the expression of IL-6 was significantly lower in the wild type animals compared to mFPR1 (p<0.05) and mFPR2-deficient mice (p<0.05). The expression of TNF-α (Fig. 4B) was significantly increased in the mFPR1 (p<0.05) and mFPR2-deficient mice (p<0.001) compared to the WT mice 3 h as well as 6 h after LPS-administration. Correlated to IL-6 gene expression also the cytokine CXCL1 displayed divergent levels of mRNA expression. At 3 h after LPS-stimulation CXCL1 has a higher expression in WT-mice compared to mFPR1^-/-^ and mFPR2^-/-^. The tendency did not reach a level of significance. At 6 h post LPS injection mFPR1-knockout and mFPR2-knockout mice displayed a significant higher expression of CXCL1 compared to wild type mice (fig. 4C).

### Altered bacterial recognition after deficiency of mFPR1 or mFPR2 is suspected

The quantitative analysis of TLR4 and TLR2 expression displayed a highly different pattern. Toll-like receptor 2 gene expression is strongly elevated in all mice strains which underwent LPS-stimulation at time point 3 h. The differences in gene expression are significant between mFPR2^-/-^ and WT mice, no significance could be found in comparison of mFPR1^-/-^ and WT mice. At 6 h post LPS injection the expression of the TLR2 gene is still induced in all mice strains and displayed a reduction compared to the 3 h time point and no significant differences among the different genotypes used in the experiment (Fig. 4D). The analysis of the Toll-like receptor 4 gene expression (Fig. 4E) showed a highly interesting pattern. WT mice displayed a significant higher induction of the TLR4-expression at 3 h after LPS-stimulation whereas mFPR1 and mFPR2-deficient mice displayed a significantly lower induction of the TLR4 gene expression (p<0.05). At 6 h post LPS injection a turnaround was visible. The mFPR1^-/-^ and mFPR2^-/-^ mice displayed a significantly higher expression of the TLR4 gene compared to WT mice at this time point (p<0.05).

### FPR-deficient liver seem to be more sensitive to cytotoxicity

**Figure 5:**
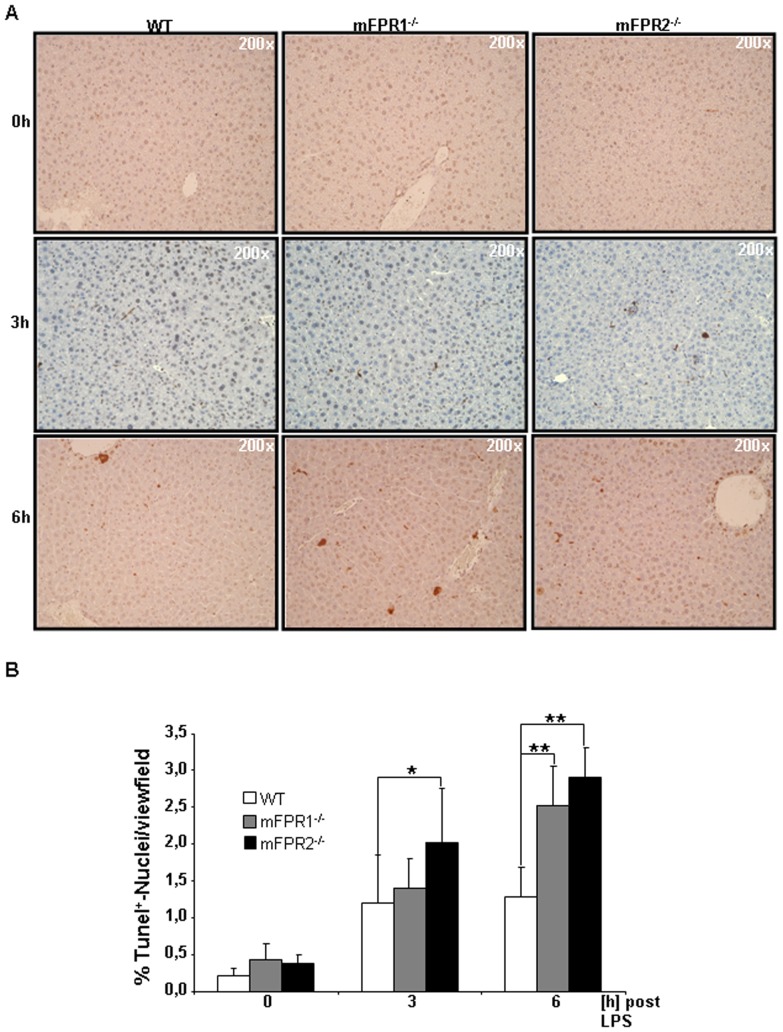
For determination of the liver injury FFPE-section were stained for DNA-strand breaks using a TUNEL-assay. Tunel^+^-cells were counted. Counterstaining was perfomed using Haematoxylin (A). Pictures were taken in 200 fold magnification. Results displaying an increase of TUNEL^+^-cells in regard to mFPR1 and mFPR2-deficiency are shown as a Graph (B). (* = p<0.05; ** = p<0.01.)

To understand whether livers lacking mFPR1 and mFPR2 were more susceptible to pro-apoptotic signalling, a TUNEL-assay was performed (Fig. 5A and B).

The analysis of the 3 h time point revealed a higher percentage of TUNEL^+^-cells in mFPR1 (1.4%) and mFPR2 deficient mice (2.03%) compared to WT mice (1.2%). At 3 h only mFPR2^-/-^ mice had a significantly higher number of TUNEL^+^-cells detectable in the liver. At the 6 h time point the amount of TUNEL^+^-cells was elevated in all mice strains. However, mFPR1^-/-^ mice (2.53%) and mFPR2^-/-^ mice (2.91%) had both significantly more apoptotic cells detectable in comparison to WT mice (1.28%) 6 h after injection of LPS (Fig. 5A and B) (WT vs mFPR1^-/-^ p<0.05; WT vs mFPR2^-/-^ p<0.01). The mFPR2^-/-^ mice itself displayed also a non-significant higher tendency for the number of TUNEL^+^-cells in comparison to the mFPR1^-/-^ mice at 3 h and 6 h post LPS administration.

### Expression profiles of murine FPRs in cholestatic liver injury in mice

**Figure 6:**
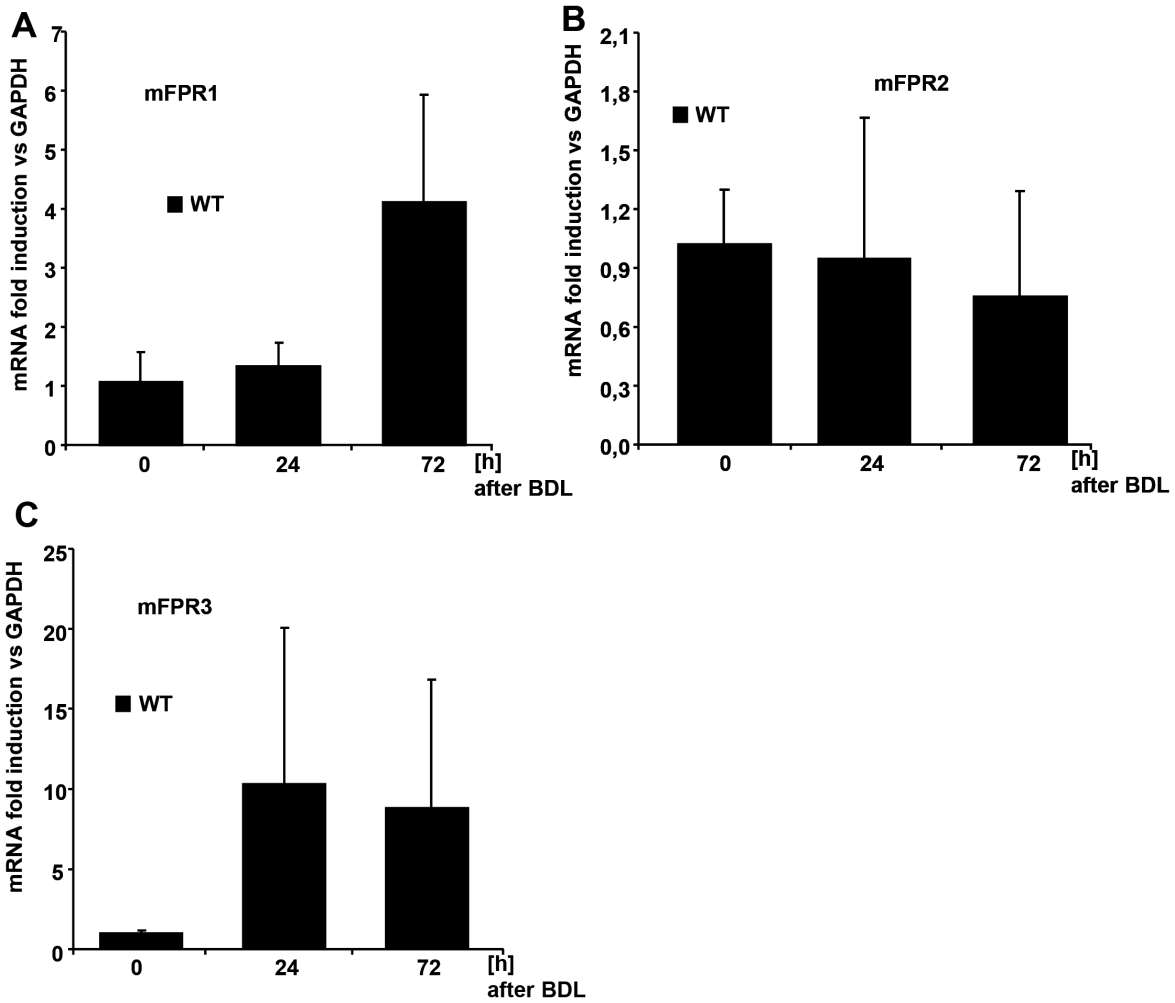
The Expression of the three mFPRs mFPR1 (A), mFPR2 (B) and mFPR3 (C) was analysed by qPCR and the gene expression was related to GAPDH. For the analysis of inflammation in the BDL-model immune cells were stained for CD11b-positivity. Graphical score for the amount of CD11b^+^-cells in the liver displays an increase over time. The cells are displayed as numbers per view field (D).

To investigate whether mFPRs are also relevant in other models of liver injury, we investigated the gene expression of formyl peptide receptors in a surgical model of cholestatic liver injury (bile duct ligation/BDL) [27]. We analysed the early time points of cholestatic liver injury 24 h and 72 h post BDL for changes in formyl peptide receptor expression (Fig. 6A to C). We were able to display an increment of mFPR1 expression at 72 h post BDL in comparison to the basal expression at time point 0 h (Fig. 6A). No increase was also detected 24 h post BDL. Interestingly the mFPR2-receptor gene displayed no increase during the early phase of cholestatic liver injury (Fig. 6B). The formyl peptide receptor 3 (mFPR3) displayed an elevated gene expression at 24 h and 72 h after BDL (Fig. 6C). The increase of mFPRs was also associated with an increase of CD11b^+^ in the livers of these mice (Figure S1A and B) during the early phase of cholestatic liver injury at 24 and 72 h post BDL.

### Compensatory liver proliferation after LPS-stimulus

**Figure 7:**
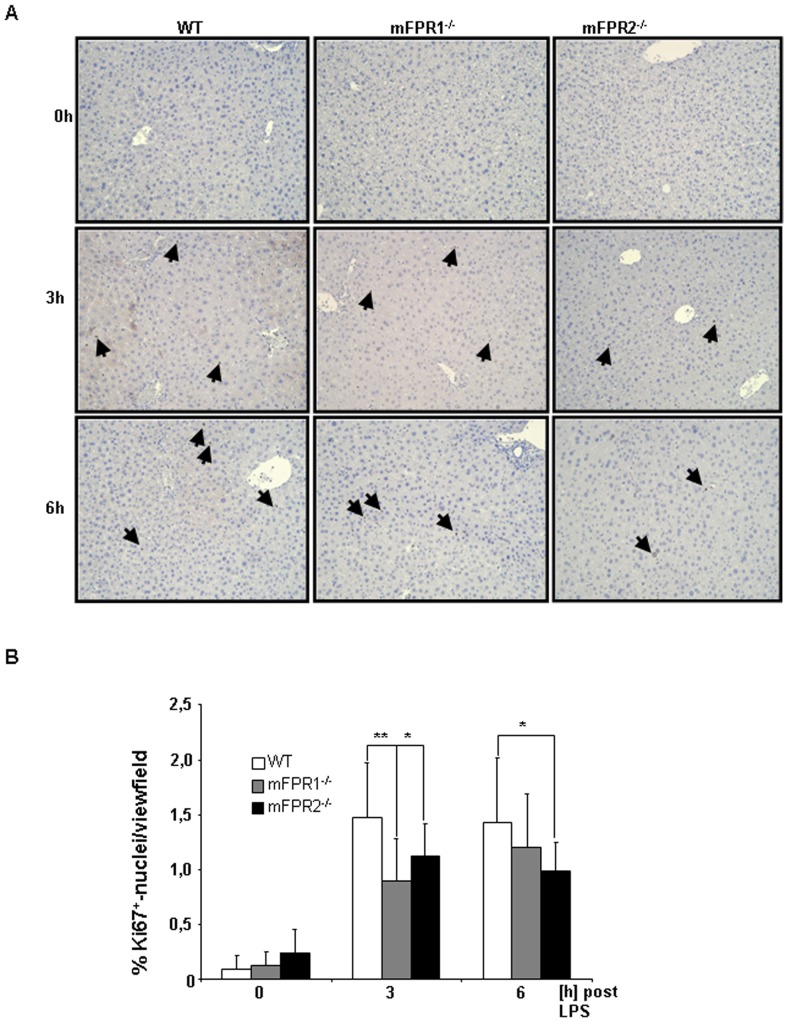
To investigate liver proliferation FFPE-sections were stained with the universal cell cycle marker Ki67. At 3^+^-nuclei were counted and analyzed as percentage of proliferative cells. Photomicrographs were taken at 200-fold and representative images are shown. Ki67^+^-nuclei are indicated by arrows (* = p<0.05; ** = p<0.01).

Liver regeneration is a response to compensate the loss of cellular mass after an injury. To understand whether mFPR1 and mFPR2 might have an impact on liver regeneration we analysed the proliferative capacity of livers lacking either mFPR1 or mFPR2. For this purpose, the ubiquitous cell cycle marker Ki67 was stained at 0 h, 3 h and 6 h post LPS stimulation. The analysis of the staining revealed a lower number of Ki67^+^-nuclei in the liver of either mFPR1 or mFPR2-deficient mice in comparison to wild type mice (Fig. 7 A and B). At 3 h mFPR1^-/-^ (0,89%) and mFPR2^-/-^ (1,19%) displayed a significantly lower number of proliferative cells compared to WT mice (1,47%) (mFPR1^-/-^ vs WT p<0.01, mFPR2^-/-^ vs WT; p<0.05). At 6 h post LPS administration the differences between WT (1,42) and mFPR1^-/-^ (1,20%) were still present but did not reached a level of significance. The significant lower proliferation of mFPR-deficient mice (0,99%) compared to WT mice was still present at this time point (p<0.05).

## Discussion

Formyl peptide receptors 1 and 2 are known to function as important mediators of chemotaxis of hematopoietic cells [28], [29]. The receptors interact with a menagerie of structurally diverse pro- and anti-inflammatory ligands associated with different diseases, including amyloidosis, Alzheimer's disease, prion disease and HIV [29]–[31]. After activation by theirs respective ligands either fMLF or Lipoxin A4, FPRs induce various effects to haematopoietic cells such as chemotaxis or release of superoxide [29], [32], [33]. Furthermore it was shown that deficiency of FPR1 in neutrophils leads to disorientation and inability to migrate to an area of injury [14] e.g. the liver. So far the knowledge about their role in parenchymal liver cells is marginal. For FPR2 it has to be pointed out, that it plays a promiscuous role. On the one hand it can interact with pro-inflammatory ligand such as fMLF and Cramp [34] on the other hand it also interacts with anti-inflammatory ligands with seem to have a more prominent effect in activation of FPR2 downstream signalling [35], [36]. Previous work about regulation of liver inflammation after an injury pointed out the importance of maintaining liver homeostasis and to avoid chronic inflammatory liver injury and in the final stages chronic liver diseases [20], [37], [38].

We compared the effect of mFPR1 and mFPR2 deficiency after LPS-stimulation, mimicking a bacterial mediated liver injury. The initial analysis of the transaminases AS and ALT (Fig. 1A and B) from the LPS treated mice strains displayed at 3 h post LPS no difference among the wild type and mFPR1^-/-^ mice. The mFPR2^-/-^ mice displayed a significant higher level of ALT in the serum. At the later 6 h time point, both, mFPR1^-/-^ and mFPR2^-/-^, displayed a significantly higher Level of ALT. For AST a slightly different pattern appears. Wild type mice had the highest levels of AST detectable in the serum compared to mFPR1 and mFPR2-deficient mice. 6 h post LPS the mFPR1 and mFPR2 knockout mice displayed significant higher levels of AST in the serum. These findings support a protective role for formyl peptide receptors during progression of LPS induced liver injury. The histological analysis after LPS-stimulation revealed a differential recruitment of immune cells in a time and genotype dependent manner. The cytokine IL-6 is not only known as a recruiting molecule for immune cells. It is described as one of the main drivers of hepatoprotection during liver injury [39], which mediates hepatoprotection against FAS-induced apoptosis [40] as well as TNF-α induced apoptosis in the liver [41]. The differential expression of IL-6, which is described as one of the most critical regulators of the immune response in the liver suggest, that mFPR1 mediated signalling is involved in the regulation of the early phase of inflammatory response in the liver and as a possible modulator of IL-6 signalling. A similar pattern is shown by the analysis of the expression of CXCL1 which correlates with the IL-6 expression [42]. At the 6h time point increased migration of immune cells to the liver of mFPR1- and mFPR2-deficient mice. The more detailed analysis of those liver infiltrating cells was done by staining FFPE-liver tissues with antibodies for myeoloid cells (CD11b) or neutrophils (Ly6G). In comparison to wild type mice monocytes and neutrophils displayed a stronger presence in the livers of FPR1^-/-^ and FPR2^-/-^ mice 6 h after LPS stimulation. Interestingly mFPR2^-/-^ mice showed a lower tendency regarding the number of Ly6G^+^-cells visible per view field. The closest explanation for this phenomenon is the link to the neutrophil recruiting cytokine CXCL1 (CXCL1) which showed the same tendency at least on mRNA level at 3 h and at 6 h post LPS-stimulation. Differential roles for mFPR1 and mFPR2 regarding immune cell homing is not excluded for granulocytes and supported by the literature [14], [43]. It was shown recently, that FPR1 regulates the anti-inflammatory response [44]. Whether this response is correlated to the IL-6 signalling remains to be investigated.

A further finding of the regulation of the anti-inflammatory response is visible for other PAMP-receptors such as TLR4 and TLR2 (Fig. 4D and E). The analysis of theirs expression by qPCR reveals an increment in mFPR1 and mFPR2-deficient animals for TLR2 3 h and 6 h post LPS. TLR4 showed a high expression in WT animals at 3 h and a significantly reduced expression in mFPR1 and mFPR2 deficient mice. On the other hand 6 h post LPS stimulation the TLR4 gene is significantly higher expressed in the mFPR1 and mFPR2-knockout mice. This leads to the conclusion that pathogen recognition might be delayed in the formyl peptide-receptor deficient mice and that both receptors contribute to this recognition and are essential for a stringent and correct procedure of this. This furthermore supports an immunomodulatory function of FPRs, which might not be exclusively provided by hematopoietic cells but also by parenchymal liver cells e.g. hepatocytes and hepatic stellate cells. In contrast to previous publications [45], the number of apoptotic cells in the liver of mFPR2^-/-^ mice was the highest of all mice strains used in this study. The anti-apoptotic capabilities of FPR2/FPRL1 in primary human neutrophils are controversially discussed. Previous observations by Nagaoka et al. revealed that FPR2 is protective together with the P2X7 receptor [46]. It was shown later that the distinct presence of Serum amyloid A (SAA), activates a protective signalling pathway which is P2X7 dependent, but FPR2/FPRL1 independent [47]. So far, the participation of mFPR2 in the regulation of the liver inflammation remains to be investigated in detail. A better explanation for the higher rate of apoptosis is the stronger expression of the pro-inflammatory and pro-apoptotic cytokine TNF-α, a cytokine with pro-apoptotic abilities. It is significantly stronger expressed in either mFPR1^-/-^ and mFPR2^-/-^ mice in comparison to WT-mice. Furthermore these findings suggest a specific anti-apoptotic signalling of mFPR2 towards TNF-α induced pro-apoptotic signalling. Stimulation experiments using a combination of fMLF and pharmacological inhibitors for p38 and MEK resulted in reduced chemotaxis, adhesion and release of superoxide by neutrophils [33] supporting a hypothesis of intracellular pathway modulation by FPRs.

The investigation of liver proliferation to compensate the loss of liver mass, displayed an impairment of regenerative capacity in mFPR1 and mFPR2-deficient mice. Both genotypes showed a lower proliferation at 3 h and 6 h post LPS-induced liver injury, suggesting critical involvement of mFPR1 and mFPR2 in liver regeneration. Recent studies of liver regeneration showed that other member of the GPCR family especially the cannabinoid type 1 receptors support these findings. Furthermore, it puts the Ca^2+-^Signalling into the focus of attention and suggests an involvement of Ca^2+^ induced signalling in the mediation of cell cycle progression [48], [49].

The analysis of the mRNA expression of mFPR1-3 in the BDL model shows an increase of mFPR1 and mFPR3 gene expression in concordance with the number of infiltrating monocytes in the liver (Fig. 6A–C, Fig S1 A&B). This finding suggests a relevant role for FPRs in the regulation of the inflammatory response of the liver after an injury stimulus. Earlier findings indicate a relevant role of mFPRs in the clearance of the sepsis [14]. A previous publication of our group linked functional signalling cascades to a pro-survival phenotype in a murine model of acute biliary injury [22]. The analysis of liver inflammation in the cholestatic liver injury model for the variation of mFPR expression at the time point 24 h and 72 h post BDL suggests that a coordinated presence and expression is important for maintaining the equilibrium of inflammation also to avoid a chronic inflammation which leads to liver fibrosis.

The consequences of FPR deficiency in an acute model of liver injury seem to be a loss of anti-apoptotic and anti-inflammatory capabilities. Our findings suggest a pro-survival, anti-inflammatory role during an acute LPS-induced liver injury. This is in concordance with previous findings suggesting a positive role for FPR during the phase of acute injury [14], [50]. How these signals are regulated by FPRs remains unclear as well as an interconnection between FPR mediated signalling pathways towards pro-inflammatory pathways such as IL-6 or pro-apoptotic pathways such as TNF-α remain to be investigated.

Furthermore, the role of FPRs in progression of chronic inflammatory liver diseases is not understood and ought to be investigated. Based on recent investigations FPR expression could also be shown on natural killer cells [43] suggest that the effects of the formyl peptide receptors is not limited to the innate inflammatory response. A more detailed analysis of the immune cell subtypes after deletion of mFPRs will show whether there is a not only a disorientation of neutrophils [14] but also a change in other myeloid and lymphoid leukocyte populations. This extends the prior findings e.g. in neuronal tissue [26] and the immune cell migration. Whether our findings show the cause of inflammation or the response only is hard to define. It is important to point out that formyl peptide receptors do play a prominent role in the mediation of the inflammatory response in the liver and that they are involved in the mediation of pathogen recognition. This results in a delayed expression of TLR4. As a consequence of this delay we also suggest a differential role for mFPR1 and mFPR2. The lack of mFPR1 leads to disruption of the early inflammatory homeostasis. This disequilibrium leads to an increased inflammatory response at the later stages of inflammation. Murine FPR2 deficiency seems to be more related to a lack of anti apoptotic and hepatoprotective function. This leads to a stronger expression of pro inflammatory cytokines which results in a higher grade of liver inflammation associated with a higher number of apoptotic cells in the liver and a reduced regenerative capacity. Furthermore, analysis regarding the influence of fMLF and its antagonists displayed a strong influence e.g. on osteogenesis [51] by regulating the differentiation of progenitor cells via the ERK-pathway. Progenitor cells also do play a critical role in the liver and their function during liver regeneration is intensively discussed [52], [53]. If fMLF also has an influence on this type of progenitor cells and by this also on the liver regeneration remains to be investigated in detail. Future investigations of our group will aim on a more detailed analysis of formyl peptide receptor signalling pathways in regard to pathogenesis of acute and chronic inflammatory liver diseases such as acute liver failure, NASH (Non-alcoholic-steato-hepatitis) and liver fibrosis.

Taken together both receptors are important to maintain a functional response to LPS induced liver injury. Furthermore our data suggests that mFPR1 and mFPR2 might also be involved in processes such as liver regeneration and might also have relevance not only during the acute liver injury, but also during chronic liver injury. Further experiments will provide prove to this hypothesis.

## Supporting Information

Figure S1 **Immunofluorescent staining for CD11b reveals an increase over time after BDL.** CD11b^+^-cells were visualized using Alexa546. Nuclei were counterstained using DAPI.
(TIF)
