# Supplementary figures and images for: Deficiency of Formyl Peptide Receptor 1 and 2 Is Associated with Increased Inflammation and Enhanced Liver Injury after LPS-Stimulation
Source: PLoS One. 2014 Jun 23;9(6):e100522. doi: 10.1371/journal.pone.0100522 (PMC4067326; doi:10.1371/journal.pone.0100522)

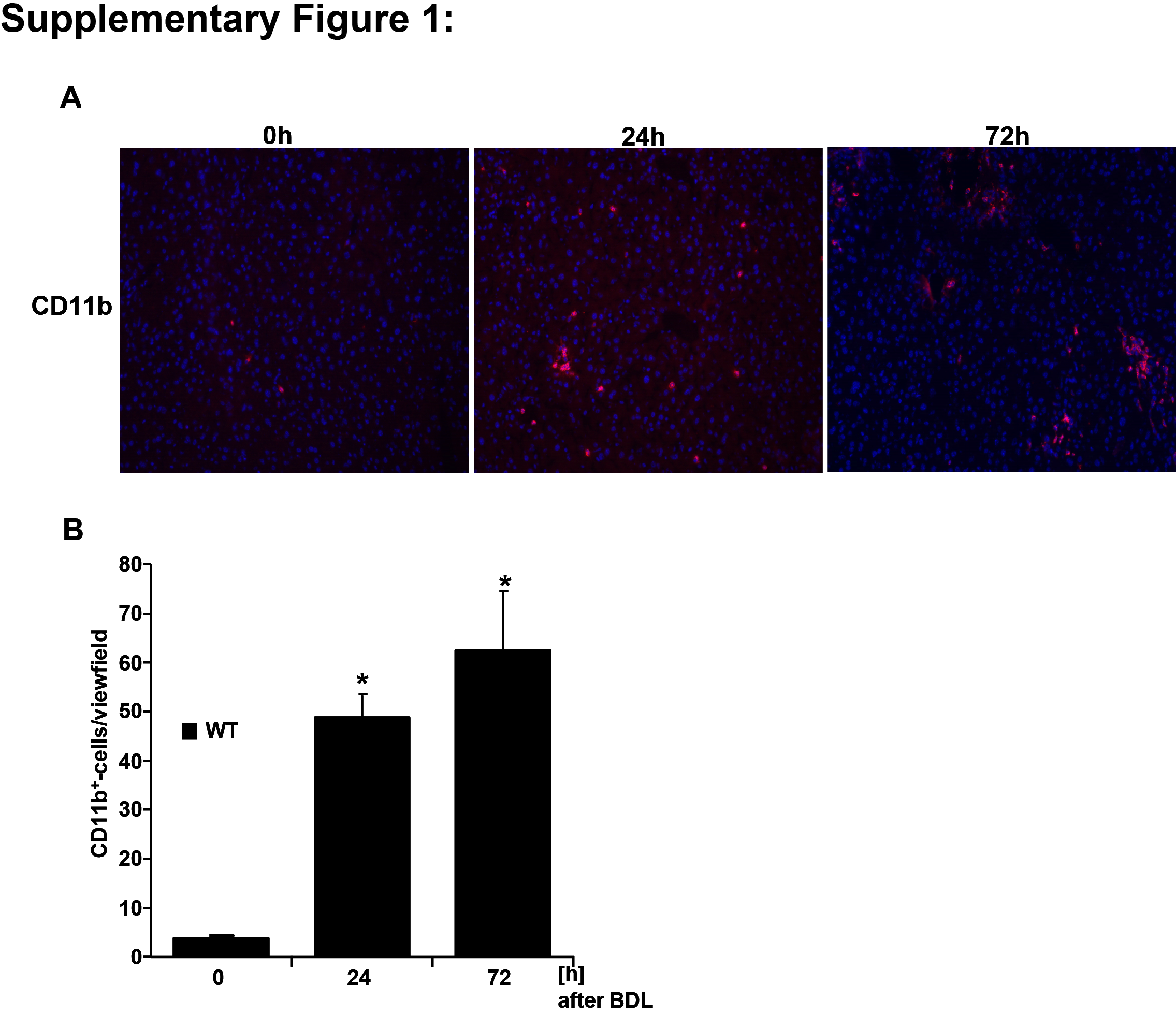

Supplement: Figure S1 — Immunofluorescent staining for CD11b reveals an increase over time after BDL. CD11b+-cells were visualized using Alexa546. Nuclei were counterstained using DAPI. (TIF) [file pone.0100522.s001.tif]
